# Eyeblink Detection in the Field: A Proof of Concept Study of Two Mobile Optical Eye-Trackers

**DOI:** 10.1093/milmed/usab032

**Published:** 2021-02-10

**Authors:** Theresa Schweizer, Thomas Wyss, Rahel Gilgen-Ammann

**Affiliations:** Monitoring Canton: Bern, Swiss Federal Institute of Sport Magglingen (SFISM), Magglingen/Macolin 2532, Switzerland; Monitoring Canton: Bern, Swiss Federal Institute of Sport Magglingen (SFISM), Magglingen/Macolin 2532, Switzerland; Monitoring Canton: Bern, Swiss Federal Institute of Sport Magglingen (SFISM), Magglingen/Macolin 2532, Switzerland

## Abstract

**Introduction:**

High physical and cognitive strain, high pressure, and sleep deficit are part of daily life for military professionals and civilians working in physiologically demanding environments. As a result, cognitive and physical capacities decline and the risk of illness, injury, or accidents increases. Such unfortunate outcomes could be prevented by tracking real-time physiological information, revealing individuals’ objective fatigue levels. Oculometrics, and especially eyeblinks, have been shown to be promising biomarkers that reflect fatigue development. Head-mounted optical eye-trackers are a common method to monitor these oculometrics. However, studies measuring eyeblink detection in real-life settings have been lacking in the literature. Therefore, this study aims to validate two current mobile optical eye-trackers in an unrestrained military training environment.

**Materials and Method:**

Three male participants (age 20.0 ± 1.0) of the Swiss Armed Forces participated in this study by wearing three optical eye-trackers, two VPS16s (Viewpointsystem GmbH, Vienna, Austria) and one Pupil Core (Pupil Labs GmbH, Berlin, Germany), during four military training events: Healthcare education, orienteering, shooting, and military marching. Software outputs were analyzed against a visual inspection (VI) of the video recordings of participants’ eyes via the respective software. Absolute and relative blink numbers were provided. Each blink detected by the software was classified as a “true blink” (TB) when it occurred in the software output and the VI at the same time, as a “false blink” (FB) when it occurred in the software but not in the VI, and as a “missed blink” (MB) when the software failed to detect a blink that occurred in the VI. The FBs were further examined for causes of the incorrect recordings, and they were divided into four categories: “sunlight,” “movements,” “lost pupil,” and “double-counted”. Blink frequency (i.e., blinks per minute) was also analyzed.

**Results:**

Overall, 49.3% and 72.5% of registered eyeblinks were classified as TBs for the VPS16 and Pupil Core, respectively. The VPS16 recorded 50.7% of FBs and accounted for 8.5% of MBs, while the Pupil Core recorded 27.5% of FBs and accounted for 55.5% of MBs. The majority of FBs—45.5% and 73.9% for the VPS16 and Pupil Core, respectively—were erroneously recorded due to participants’ eye movements while looking up, down, or to one side. For blink frequency analysis, systematic biases (±limits of agreement) stood at 23.3 (±43.5) and −4.87 (±14.1) blinks per minute for the VPS16 and Pupil Core, respectively. Significant differences in systematic bias between devices and the respective VIs were found for nearly all activities (*P* < .05).

**Conclusion:**

An objective physiological monitoring of fatigue is necessary for soldiers as well as civil professionals who are exposed to higher risks when their cognitive or physical capacities weaken. However, optical eye-trackers’ accuracy has not been specified under field conditions—especially not in monitoring fatigue. The significant overestimation and underestimation of the VPS16 and Pupil Core, respectively, demonstrate the general difficulty of blink detection in the field.

## INTRODUCTION

In physiologically demanding operational environments, such as military service, fatigue is common. Soldiers are constantly exposed to a high level of mental and physical strain, high pressure, sustained wakefulness, and a high operational tempo, i.e., high speed and intensity of actions. In addition, soldiers’ sleep quantity and quality are affected by uncomfortable sleeping environments, night exercises, and sleep deprivation over long periods.^1–4^ Soldiers’ demanding training sessions and operations, as well as their insufficient quality and quantity of sleep, have been recognized as critical variables accompanying a significant reduction in their ability to perform cognitive or physical activities.^5–8^ Indeed, several authors have emphasized that this suboptimal physiological state affects individuals’ cognitive and physical readiness and increases their risk of illness, injury, or accidents.^7,9–11^ Notably, a decline in cognitive and physical performance can occur before an individual’s self-awareness of being tired and, therefore, can enhance the risk of unfortunate outcomes.^6^ Moreover, an individual’s psychological state (e.g., motivation) might influence their perception of their actual level of fatigue.^5^ Consequently, the objective, individual, and real-time monitoring of fatigue has become a growing concern in military occupations. Real-time physiological monitoring could provide necessary information about individuals’ health and performance status and, therefore, help to alert soldiers or their commander about their state before their fatigue level compromises their own and others’ safety.^12^

Two decades ago, Morris and Miller (1996)^13^ reported that performance decrements due to changes in fatigue could be assessed through oculometrics. Since then, various studies have investigated oculometrics’ sensitivity to the development of fatigue in different tasks—such as computer work, driving, flying, and air traffic control.^14–18^ Oculometrics have been perceived as a reflection of underlying neural mechanisms that can be regarded as promising biomarkers for the early detection of fatigue.^17–19^ Among the possible oculometrics in such uses, eyeblinks are the most predominant ocular event in the literature to monitor fatigue because they are easily observable and known to correlate with the development of fatigue.

To monitor soldiers’ fatigue during daily service, the only possible eye-tracking devices are mounted on soldiers’ heads. One of the most common methods to track eye movements is a mobile optical eye-tracking system.^20^ Previously, testings in a laboratory or controlled situations revealed that a few challenges—such as movements and lighting conditions—must be considered when using optical eye-trackers.^21,22^ Although eye-tracking glasses are meant to be implemented in the field and fatigue should be monitored in real life, the literature has lacked validity and feasibility studies. Therefore, this study aims to validate eyeblink detection for the VPS16 and Pupil Core optical eye-trackers during normal, unrestrained military training in the field.

## METHOD

### Participants

Three healthy male participants (age: 20.0 ± 1.0 years; height: 177.9 ± 3.1 cm; weight: 78.2 ± 12.3 kg) of the Special Forces Command Grenadiers of the Swiss Armed Forces gave their written informed consent to participate in this study. Before data collection, local ethical approval was granted by the Institutional Review Board of the Swiss Federal Institute of Sport in Magglingen (Nr: 2019/096). No participant had any visual disturbance or weakness that made wearing glasses or contact lenses necessary, nor did participants take any systematic medication likely to provoke dry eyes, have a history of eye pathology, or have any subjective eye complaint. Medical professionals conducted extended health screening of every potential Swiss Armed Forces member during the recruiting process, 3-12 months before the beginning of their basic military training.

### Study Design

This study was an observational, nonexperimental study analyzing eyeblink data of participants in a military real-world operational setting. Three eye-tracking devices were tested in terms of blink accuracy: Two VPS16s (Viewpointsystem GmbH, Vienna, Austria) and one Pupil Core (Pupil Labs GmbH, Berlin, Germany). These numbers and these types of devices were chosen due to availabilities and current technology developments. The measurements took place on two separate days in January 2020. In total, four measurements were done, two on each day allowing for a data collection of four different activities: Healthcare education (morning), orienteering (afternoon), shooting training (morning), and military marching training (afternoon). The four activities were chosen according to participants’ daily military training schedule in order to have four different activities in two different sunlight conditions: In the early morning before sunrise and in the afternoon under sunny conditions. The three participants wearing the eye-tracking glasses joined the other soldiers and followed their superiors’ instructions for the measurement period of 1 h. After each measurement, the participants stated their mean rate of perceived physical and mental exertion using the relative values exposed by Chowdhury et al. (2019),^23^ classified into three levels (low: <55, moderate: 55-70, and high: 71-100). The hourly mean values for sunlight, rain, temperature, and humidity were taken at the nearest location to the military garrison (Federal Office of Meteorology and Climatology, MeteoSchweiz).

### Instruments

Two different mobile optical eye-tracking devices were evaluated. The first of these devices was the VPS16 binocular wearable eye-tracking system, which uses two small eye-cameras with infrared light. The eye-cameras recorded data with a sample rate of 25 Hz. A world camera captured scenes in front of participants. The VPS16 was connected via a cable to a portable smart unit placed in the front pocket of participants’ jackets, which analyzed the data from the eye-tracking wearable. The initial stepwise calibration for the VPS16 with the smart unit was conducted according to the manufacturer’s instructions to determine the best-fitting nose pad and calibrate the eye gaze. After measurement, the data were transferred to a software application called “Fact Finder” (Version 2016, Viewpointsystem, Vienna, Austria), which visualized and analyzed participants’ eyeblinks. To detect participants’ pupils, an algorithm searched for the largest possible black area and then calculated an ellipse surrounding this area. A blink was counted when 50% of the pupil was covered.

The second of these devices, the Pupil Core binocular wearable eye-tracking headset, used with the Pupil Core open-source eye-tracking software (Pupil Labs GmbH, Berlin, Germany), has two eye-cameras on the side of its frames. These eye-cameras recorded data with a sample rate of 120 Hz. During measurements, the Pupil Core headset was connected via a cable to a mobile phone that recorded eye movements using the Pupil Capture software (Version 1.21.5, Pupil Labs, Berlin, Germany). To calibrate the Pupil Core device, participants had to move their wide-open eyes around for a few seconds so that the algorithm could detect their pupils. These recordings were transferred to the Pupil Player software (Version 1.21.5, Pupil Labs, Berlin, Germany) to analyze participants’ eyeblinks. The pupil detection algorithm estimated the approximate center of the eyeball’s rotation and a three-dimensional (3D) pose of the pupil (modeled as a 3D disc). Further, this algorithm detected the pupil by searching for the darkest region and creating an ellipse around this region. For the VPS16, the algorithm recorded a blink when this dark, round area (i.e., the pupil) was more than 50% covered.

### Data Processing

This study analyzed minutes 0-10 of each measurement and military activity. Overall, 12 sequences of 10 min (eight for the VPS16 and four for the Pupil Core) were analyzed, corresponding to a total of 80 min and 40 min of analyzed data, respectively, for the VPS16 and Pupil Core.

**FIGURE 1.**
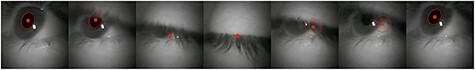
Example of a true blink (TB; i.e., a blink that was recorded by the software and occurred in the visual inspection [VI] at the same time). This example is from the Pupil Core device. The ellipse around the pupil disappears when the pupil is more than 50% covered, and this event is counted as an eye blink.

After each measurement, data were uploaded to the respective software and exported as a Microsoft Excel document (Microsoft Office 2016, Microsoft Corporation, Redmond, WA). The respective software provided an output for the detected eyeblinks as well as each blink’s start and end times. Both devices also provided video recordings of the eyes during measurements. These video recordings were visually inspected in slow motion (×0.25 the original speed), and blink events were identified. The resulting data were considered as this study’s reference data and, subsequently, named “visual inspection” (VI). The VI was synchronized with the respective software’s outputs. Each blink detected by the software was examined and classified as a “true blink” (TB), “false blink” (FB), or “missed blink” (MB). The blinks were classified as TBs when they were recorded by the respective software at the same time as in the VI (Fig. 1). They were classified as FBs when they occurred in the software but not in the VI, and they were classified as MBs when the software failed to detect the blinks compared to the VI.

**FIGURE 2.**
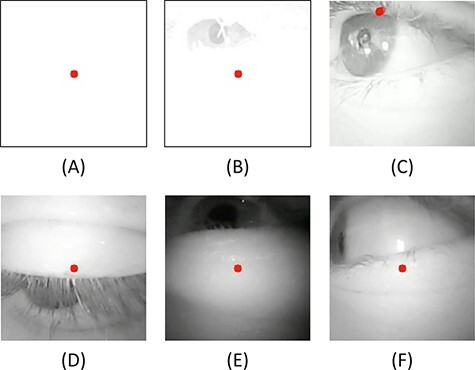
Six situations that caused erroneous blink detection by the software. Examples B-F were categorized as “false blinks”: (A) NO_im_ = No image was available because of extreme sunlight, (B) sunlight (e.g., sunlight shining onto the eye), (C) movements (e.g., running), (D) lost pupil (looking downward), (E) lost pupil (looking upward), (F) lost pupil (looking to the side).

The FBs were further examined for the causes of the incorrect recordings by the respective software and divided into four categories:

1. “Sunlight”: The sun was shining right into the camera or onto the eye, altering the visibility of the eye or the pupil. The image of the eye did not disappear completely (Fig. 2B).
2. “Movements”: The video quality was altered because of dynamic head movements or tremors during movements (e.g., during running; Fig. 2C).
3. “Lost pupil”: The pupil was covered because the participant was looking extremely downward, upward, leftward, or rightward or the pupil was covered by the eyelashes (Fig. 2D-F).
4. “Double-counted”: The software multiplied the actual TB by two or more.

When the video recording image turned completely white because of extreme sunlight shining on the eye-cameras, it was classified as “No image” (NO_im_) since the eye was no longer visible, either for the software algorithm or for the VI (Fig. 2A).

### Statistical Analysis

Q-Q plots and the Shapiro–Wilk test for normality (*P *> .05) confirmed a normal data distribution. Descriptive statistics were used to explore participants’ anthropometrics and the recorded data. Further, mean eyeblinks per minute (i.e., blink frequency), mean absolute error (MAE), and mean absolute percentage error (MAPE) were calculated. Measurement accuracies are presented as the absolute differences and systematic biases between each device and its respective VI. Limits of agreement (LoA) were calculated using the SDs of the differences, multiplied by 1.96.^24^ Measurement agreements between the two devices (VPS16 and Pupil Core) and the VI were investigated using *t*-tests. The level for accepting statistical significance was set at *P *< .05 for all analyses. All statistical calculations were conducted using Microsoft Excel 2016 for Windows (Microsoft Corporation, Redmond, WA) and IBM SPSS Statistics 25 for Windows (IBM Corporation, Armonk, NY,).

## RESULTS

The four activities were perceived as low to moderate in terms of physical and mental load, with average values of 50.13 ± 19.01 and 47.13 ± 16.22 (scaled from 0 to 100 and classified according to Chowdhury et al., 2019^23^), respectively. Sunlight reached 0% during morning activities and 100% during afternoon activities. Temperatures varied from −1°C to 8°C, and humidity varied from 48% to 88%.

### Missing Data and No_im_

For the Pupil Core device, one entire measurement (orienteering activity) could not be analyzed because of a displacement of the glasses or the eye-cameras, such that the eye-camera lost sight of participants’ pupils. This complete activity was not considered in the following analysis. Further, 458 seconds resulted in NO_im_, representing 25.4% of the analyzed data. Therefore, the following analysis was conducted using 23 min (56%) of data for the Pupil Core device. For the VPS, 100% of the data were analyzed.

### Total Number of Examined Eyeblinks

In total, the VI assessed 2,180 blinks for the two VPS16 devices and 272 blinks for the Pupil Core device. The software for the two VPS16 devices together recorded 4,047 blinks, and the software for the Pupil Core device recorded 167 blinks. Of these total blinks, 1,994 blinks (49.3%) and 121 blinks (72.5%) were TBs for the VPS16 and Pupil Core, respectively (Table I). The remaining 2,053 (50.7%) and 46 (27.5%) blinks were FBs for the VPS16 and Pupil Core, respectively. Compared to the VI, the VPS16 counted 186 (8.5%) MBs and the Pupil Core counted 151 (55.5%) MBs.

**TABLE I.** Descriptive Results of the VPS16 and Pupil Core Devices Compared to the Visual Inspection (VI) in Absolute and Relative Numbers

Activity	Total blinks recorded		TB	FB	MB
	*N*		*N*(%)	*N*(%)	*N*(%)
	VI	VPS16			
Healthcare education	408	746	347 (46.5)	399 (53.5)	61 (15.0)
Orienteering	724	1,450	680 (46.9)	770 (53.1)	44 (6.1)
Shooting training	504	761	441 (58.0)	320 (42.0)	63 (12.5)
Military marching training	544	1,090	526 (48.3)	564 (51.7)	18 (3.3)
Overall	2,180	4,047	1,994 (49.3)	2,053 (50.7)	186 (8.5)
	VI	Pupil Core			
Healthcare education	69	52	34 (65.4)	18 (34.6)	35 (50.7)
Orienteering	–	–	–	–	–
Shooting training	130	104	81 (77.9)	23 (22.1)	49 (37.7)
Military marching training	73	11	6 (54.5)	5 (45.5)	67 (91.8)
Overall	272	167	121 (72.5)	46 (27.5)	151 (55.5)

The numbers of blinks are given in absolute numbers (*N*) and in relative numbers (%) compared to the VI. The entire orienteering-activity data are missing for the Pupil Core device.

Abbreviations: FB, false blinks; MB, missed blinks; TB, true blinks; VI, visual inspection.

### False Blinks

The majority of FBs—45.5% and 73.9% for the VPS16 and Pupil Core, respectively—were caused by participants looking up, down, left, or right, such that the algorithm could no longer detect the full size of the pupil and, therefore, falsely counted a blink (Fig. 2D-F).

For the VPS16, sunlight caused 20.1% (Fig. 2B) of total FBs, while movements caused 12.3% (Fig. 2C) and double-counted blinks caused 22.2%. For the Pupil Core, sunlight caused 10.9% (Fig. 2B) of total FBs, while movements caused 15.2% (Fig. 2C).

### Blink Frequency

Overall, the mean eyeblinks reported were 50.6 ± 27.3 versus 27.3 ± 11.3 blinks per minute for the VPS16 compared to the VI and 7.5 ± 5.3 versus 12.4 ± 7.2 blinks per minute for the Pupil Core compared to the VI (Table II). Poor agreement between the devices’ output and the VI resulted in a high systematic bias and high limits of agreements (Table II). Significant differences (*P* ≤ .05) between the VPS16, the Pupil Core, and their respective VIs were found for all activities except healthcare education using the Pupil Core.

**TABLE II.** Measurement Agreement Between the VPS16 and Pupil Core and Their Respective Visual Inspections (VIs) Regarding Blink Frequency

Activity	Average blinks per minute		MAE (MAPE)	Systematic bias ± LoA	Minutes analyzed
	VI	VPS16			
Healthcare education	20.4 ± 8.9	37.3 ± 20.1	17.2 (105.4)	16.9** ± 38.4	20
Orienteering	36.2 ± 12.8	72.5 ± 31.9	36.3 (97.1)	36.3** ± 48.1	20
Shooting training	25.2 ± 7.7	38.1 ± 16.6	13.8 (56.3)	12.9** ± 27.2	20
Military marching training	27.2 ± 9.5	54.1 ± 23.2	27.3 (137.6)	27.3** ± 44.5	20
Overall	27.3 ± 11.3	50.6 ± 27.3	23.6 (99.1)	23.3** ± 43.5	80
	VI	Pupil Core			
Healthcare education	6.9 ± 5.7	5.2 ± 4.8	3.9 (53.7)	−1.7 ± 10.1	10
Orienteering	–	–	–	–	–
Shooting training	14.8 ± 4.1	12.0 ± 2.9	3.4 (22.5)	−2.8* ± 5.4	9
Military marching training	20.5 ± 5.7	3.0 ± 2.6	17.5 (86.9)	−17.5* ± 8.7	4
Overall	12.4 ± 7.2	7.5 ± 5.3	6.1 (47.2)	−4.9* ± 14.1	23

Average blinks per minute are presented as mean ± SD.

Abbreviations: LoA, limits of agreement; MAE, mean absolute error; MAPE, mean absolute percentage error; VI, visual inspection.

* *P* ≤ .05;

** *P* ≤ .001.

## DISCUSSION

This study’s goal was to evaluate the VPS16 and Pupil Core eye-tracking devices in monitoring eyeblinks during basic, unrestrained military training under field conditions. Both devices’ eyeblink detection accuracies were insufficient for almost all the analyzed activities. The VPS16 exceedingly overestimated the numbers of eyeblinks, whereas the Pupil Core exceedingly underestimated the numbers of eyeblinks. In all measurements, the VPS16 and Pupil Core recorded 50.7% and 27.5% of FBs, respectively, and counted 8.5% and 55.5% of MBs, respectively. Similarly high missing rates and significant systematic biases were recently highlighted by Ehinger et al. (2019)^25^ using the Pupil Core.

In the present study, even the lowest MAPE during shooting training resulted in more than 20% eyeblink detection errors. The highest MAPE was observed during military marching training for both devices, at 137.6% for the VPS16 and 86.9% for the Pupil Core, clearly demonstrating a lack of measurement accuracy. Also, during low-physical-intensity exercises—such as walking, light running, or merely standing in sunny environments—countless errors in blink detection were observed. A waste majority of FBs occurred when the eye moved only a little too far up, down, right, or left. Our findings aligned with the results of Tonsen et al. (2016),^22^ who mentioned the difficulty of pupil detection in realistic day-to-day environments. Further, challenges and difficulties in maintaining ecological validity under real-world conditions—particularly in bright-light and movement conditions—have been reported.^21,26^ These findings may explain the low numbers of related studies conducted in real-life conditions. These general difficulties in real-life blink detection within field environments highlight the need for algorithm and hardware improvements.

Fatigue is an important health and safety risk factor—especially in physically or cognitively demanding occupations, such as military services. Monitoring real-time physiological bioindicators of fatigue could present a solution to preventing injuries or accidents due to a lack of attention or readiness. However, eye-tracking glasses’ lack of validity must first be resolved so that fatigue can be analyzed using this technology—particularly when a research project’s final goal is to determine individual fatigue levels in real time. For now, eye-tracking glasses’ accuracy is not established, and their application cannot be recommended.

## LIMITATIONS

A limitation of this study is its VI and labeling of FBs. In a few situations, the cause of an FB was not 100% clear and more than one label could have been involved. For example, a participant was running, which caused the wearable device to move (= “movements”) and look down (= “pupil lost”) at the same time. Furthermore, sunlight could have hidden other FB causes. Indeed, sunlight was always considered the first FB cause when it was shining onto participants’ eyes or eye-cameras because image quality decreased, making the identification of other causes difficult. A second limitation of this study is the small sample size. Only three participants have been wearing the eye-tracking glasses during the four different activities. However, the collected data points (i.e., the analyzed eyeblinks) represent a sufficiently large amount of data to be able to evaluate the accuracy of these devices under field conditions.

## CONCLUSION

This study highlighted optical eye-tracking glasses’ inaccuracy as well as their challenges and actual limitations in unconstrained field conditions. Objective physiological monitoring of fatigue is necessary for soldiers as well as other civil professionals who are exposed to higher risks if their attention is limited or reduced. However, technical and analytical improvements must be applied to eye-tracking systems before an accurate, feasible implementation in the field. For now, testing optical eye-tracking devices’ accuracy is recommended before implementing them in real-life conditions—even for light-intensity activities or tasks involving only small movements.
